# 2-[2-(4-Methoxyphenyl)-2,3-dihydro-1*H*-1,5-benzodiazepin-4-yl]phenol

**DOI:** 10.1107/S1600536809052258

**Published:** 2009-12-09

**Authors:** Yvon Bibila Mayaya Bisseyou, Ané Adjou, Yapi Marcellin Yapo, Guy Euloge Bany, R. C. A. Kakou-Yao

**Affiliations:** a Laboratoire de Cristallographie et Physique Moléculaire, UFR SSMT, Université de Cocody, 22 BP 582 Abidjan 22, Côte d’Ivoire; b Laboratoire de Chimie Organique, UFR SSMT, Université de Cocody, 22 BP 582 Abidjan 22, Côte d’Ivoire

## Abstract

In the structure of title compound, C_22_H_20_O_2_N_2_, the 11-membered benzodiazepine ring system adopts a distorted boat conformation. The benzene ring of this system forms dihedral angles of 89.69 (12) and 48.82 (12)° with those of the phenol and methoxy­phenyl substituents, respectively. The dihedral angle between the benzene rings is 49.61 (11)°. An intra­molecular O—H⋯N hydrogen bond generates an *S*(6) ring.

## Related literature

For the biological activity of heterocyclic scaffolds containing nitro­gen atoms, see: MacDonald (2002 ▶); Gringauz (1999 ▶); Albright *et al.* (1998 ▶); Rahbaek *et al.* (1999 ▶). For related structures, see: Ravichandran *et al.* (2009*a* ▶,*b* ▶,*c* ▶,*d* ▶). For puckering parameters, see: Cremer & Pople (1975 ▶). For hydrogen-bond motifs, see: Bernstein *et al.* (1995 ▶). For the weighting scheme, see: Prince (1982 ▶); Watkin (1994 ▶).
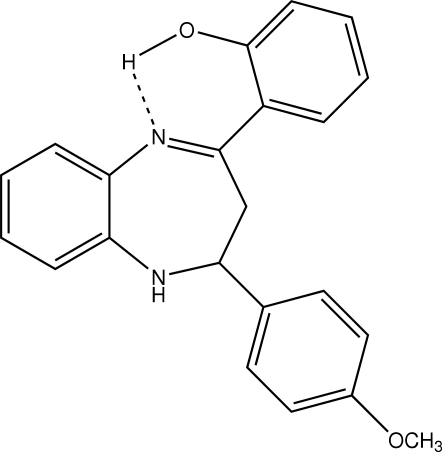


## Experimental

### 

#### Crystal data

C_22_H_20_N_2_O_2_
*M* *_r_* = 344.41
Monoclinic, 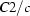

*a* = 27.5064 (5) Å
*b* = 7.3811 (2) Å
*c* = 19.5038 (4) Å
β = 117.699 (2)°
*V* = 3506.02 (15) Å^3^
*Z* = 8
Mo *K*α radiation
μ = 0.08 mm^−1^
*T* = 223 K
0.30 × 0.20 × 0.15 mm

#### Data collection

Nonius KappaCCD diffractometer
19187 measured reflections
2507 independent reflections
2836 reflections with *I* > 3σ(*I*)
*R*_int_ = 0.06

#### Refinement

*R*[*F* ^2^ > 2σ(*F* ^2^)] = 0.055
*wR*(*F* ^2^) = 0.065
*S* = 1.04
2507 reflections
235 parameters
H-atom parameters constrained
Δρ_max_ = 0.25 e Å^−3^
Δρ_min_ = −0.25 e Å^−3^

### 

Data collection: *COLLECT* (Nonius, 2001 ▶); cell refinement: *DENZO*/*SCALEPACK* (Otwinowski & Minor, 1997 ▶); data reduction: *DENZO*/*SCALEPACK*; program(s) used to solve structure: *SIR92* (Altomare *et al.*, 1994 ▶); program(s) used to refine structure: *CRYSTALS* (Betteridge *et al.*, 2003 ▶); molecular graphics: *ORTEP-3* (Farrugia, 1997 ▶); software used to prepare material for publication: *CRYSTALS*.

## Supplementary Material

Crystal structure: contains datablocks global, I. DOI: 10.1107/S1600536809052258/bq2181sup1.cif

Structure factors: contains datablocks I. DOI: 10.1107/S1600536809052258/bq2181Isup2.hkl

Additional supplementary materials:  crystallographic information; 3D view; checkCIF report

## Figures and Tables

**Table 1:** Hydrogen-bond geometry (Å, °)

*D*—H⋯*A*	*D*—H	H⋯*A*	*D*⋯*A*	*D*—H⋯*A*
O1—H11⋯N1	0.87	1.74	2.523 (3)	148

**Fig. 1.**
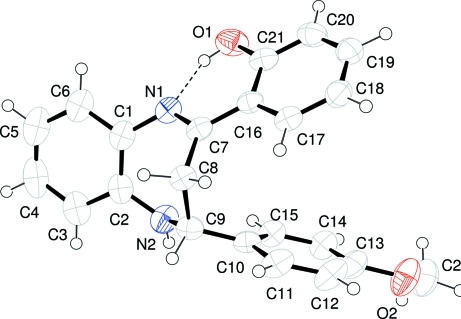
The molecular structure of the title compound and the atomic numbering scheme. Displacement ellipsoids are drawn at the 50% probability level. Dashed lines indicate hydrogen bonds.

## supplementary crystallographic information

### Comment

Heterocyclic scaffolds containing nitrogen atoms have received great attention in organic and medicinal chemistry because of their broad range of beneficial biological properties. These heterocyclic compounds such as benzodiazepines exhibit bioactive profile including anticonvulsant (MacDonald, 2002), hypnotic (Gringauz, 1999) and vasopressin antagonists (Albright *et al.*, 1998) activities. They are also used for treatment of gastrointestinal and central nervous system (*CNS*) disorder (Rahbaek *et al.*, 1999). As part of continuing work on heterocyclic compounds biologically active, we have synthesized new benzodiazepine derivative in order to explore the effects of substituents on activity and scaffold conformation of this compound class. In this paper, we present molecular structure of the title compound. The molecular structure of title compound is shown in Fig. 1. The benzodiazepine ring system adopts a distorted boat conformation as shown in the recent studies related to benzodiazepine derivatives (Ravichandran *et al.*, 2009*a*,*b*,*c*,*d*). The puckering parameters (Cremer & Pople, 1975) for this eleven-membered benzodiazepine ring system are: Q_2_ = 1.087 (3) Å,Q_3_ = 0.654 (3) Å,φ_2_ = 320.74 (4)° and φ_3_ = 26.7 (2)°. The benzene ring of this system forms dihedral angles of 89.69 (12)° and 48.82 (12)° with the phenyl rings of phenol and methoxy-phenyl fragments respectively which make them dihedral angle of 49.61 (11)°. Furthermore, there is in this structure the presence of O—H···N intra-molecular hydrogen bond, which generates an S (6) graph set motif (Bernstein *et al.*, 1995).

### Experimental

To a solution of 1-(2-hydroxyphenyl)-3-(*p*-tolyl) propenone (1.3 g, 5.4 mmol) and 1, 2-diaminobenzene in anhydrous ethanol (20 ml), was added triethylamine (6 ml, 32.4 mmol). The reaction mixture was stirred under shelter from the light for 24 h. The resulting mixture was cooled at room temperature then kept in the freezer all night long. The precipitate was then filtered and purified by chromatography silica gel. Elution solvent: hexane/ethyl acetate (90/10). We obtained yellow single crystals of title compound with a yield of 56% (m.p.: 413–415 K; Rf: 1/2, hexane/ethyl acetate: 80/20).

### Refinement

The H atoms were all located in a difference of Fourier map. They were all initially refined with soft restraints on the bond lengths and angles to regularize their geometry (C—H in the range 0.95–0.97 Å, O—H = 0.87 Å, N—H = 0.88 Å and *U*_iso_(H)in the range 1.2–1.7 times *U*_eq_ of the parent atom), after which their positions were refined with riding constraints.

### Crystal data

**Table:**

C_22_H_20_N_2_O_2_	*F*(000) = 1456
*M**_r_* = 344.41	*D*_x_ = 1.305 Mg m^−^^3^
Monoclinic, *C*2/*c*	Melting point = 413–415 K
Hall symbol: -C 2yc	Mo *K*α radiation, λ = 0.71073 Å
*a* = 27.5064 (5) Å	Cell parameters from 19187 reflections
*b* = 7.3811 (2) Å	θ = 0–0°
*c* = 19.5038 (4) Å	µ = 0.08 mm^−^^1^
β = 117.699 (2)°	*T* = 223 K
*V* = 3506.02 (15) Å^3^	Block, yellow
*Z* = 8	0.30 × 0.20 × 0.15 mm

### Data collection

**Table:**

Nonius KappaCCD diffractometer	*R*_int_ = 0.06
graphite	θ_max_ = 29.1°, θ_min_ = 1.7°
φ and ω scans	*h* = −37→32
19187 measured reflections	*k* = −10→10
2507 independent reflections	*l* = −25→25
2836 reflections with *I* > 3σ(*I*)	

### Refinement

**Table:**

Refinement on *F*^2^	Primary atom site location: structure-invariant direct methods
Least-squares matrix: full	Hydrogen site location: inferred from neighbouring sites
*R*[*F*^2^ > 2σ(*F*^2^)] = 0.055	H-atom parameters constrained
*wR*(*F*^2^) = 0.065	Method, part 1, Chebychev polynomial, (Watkin, 1994, *P*rince, 1982) [*w*eight] = 1.0/[A_0_*T_0_(x) + A_1_*T_1_(x) ··· + A_n-1_]*T_n-1_(x)] where A_i_ are the Chebychev coefficients listed belo*w* and x = *F* /*F*max Method = Robust Weighting (*P*rince, 1982) W = [*w*eight] * [1-(delta*F*/6*sigma*F*)^2^]^2^ A_i_ are: 76.3 80.0 28.8 -10.0 -11.5
*S* = 1.04	(Δ/σ)_max_ = 0.0004
2507 reflections	Δρ_max_ = 0.25 e Å^−^^3^
235 parameters	Δρ_min_ = −0.25 e Å^−^^3^
0 restraints	

### Fractional atomic coordinates and isotropic or equivalent isotropic displacement parameters (Å^2^)

**Table:**

	*x*	*y*	*z*	*U*_iso_*/*U*_eq_	
O1	0.93812 (7)	0.1834 (2)	0.28604 (9)	0.0613	
O2	0.74005 (6)	0.9744 (2)	0.19252 (11)	0.0715	
N1	0.92187 (7)	0.2773 (2)	0.15267 (10)	0.0441	
N2	0.82826 (7)	0.4219 (3)	0.02002 (10)	0.0518	
C1	0.90766 (9)	0.2197 (3)	0.07668 (13)	0.0465	
C2	0.86133 (9)	0.2877 (3)	0.01157 (13)	0.0481	
C3	0.84587 (10)	0.2063 (4)	−0.06017 (14)	0.0600	
C4	0.87638 (12)	0.0637 (4)	−0.06710 (17)	0.0694	
C5	0.92265 (12)	0.0009 (4)	−0.00312 (18)	0.0690	
C6	0.93752 (10)	0.0773 (3)	0.06788 (16)	0.0572	
C7	0.92184 (7)	0.4477 (3)	0.16870 (12)	0.0385	
C8	0.91134 (8)	0.5878 (3)	0.10725 (12)	0.0404	
C9	0.85020 (8)	0.6032 (3)	0.04950 (12)	0.0435	
C10	0.81802 (8)	0.6940 (3)	0.08532 (11)	0.0405	
C11	0.82500 (9)	0.8789 (3)	0.10081 (14)	0.0547	
C12	0.79940 (9)	0.9683 (3)	0.13693 (15)	0.0584	
C13	0.76448 (8)	0.8738 (3)	0.15756 (14)	0.0526	
C14	0.75569 (9)	0.6925 (3)	0.14123 (13)	0.0509	
C15	0.78302 (8)	0.6032 (3)	0.10597 (12)	0.0475	
C16	0.93285 (8)	0.4968 (3)	0.24729 (12)	0.0384	
C17	0.93531 (8)	0.6780 (3)	0.27066 (12)	0.0442	
C18	0.94419 (8)	0.7245 (3)	0.34386 (13)	0.0506	
C19	0.95159 (9)	0.5901 (3)	0.39714 (13)	0.0532	
C20	0.95014 (9)	0.4115 (3)	0.37696 (13)	0.0532	
C21	0.94043 (8)	0.3622 (3)	0.30257 (13)	0.0455	
C22	0.69528 (10)	0.8907 (4)	0.19955 (17)	0.0766	
H82	0.9309	0.5495	0.0775	0.0489*	
H81	0.9244	0.7079	0.1309	0.0491*	
H191	0.9568	0.6222	0.4495	0.0668*	
H91	0.8471	0.6809	0.0055	0.0540*	
H111	0.8489	0.9442	0.0849	0.0673*	
H141	0.7308	0.6258	0.1547	0.0624*	
H151	0.7763	0.4730	0.0942	0.0604*	
H201	0.9565	0.3167	0.4133	0.0656*	
H51	0.9445	−0.0943	−0.0083	0.0953*	
H171	0.9305	0.7735	0.2334	0.0558*	
H181	0.9460	0.8507	0.3583	0.0644*	
H21	0.7927	0.4133	−0.0098	0.0665*	
H41	0.8660	0.0108	−0.1167	0.0911*	
H121	0.8062	1.0951	0.1497	0.0723*	
H31	0.8135	0.2496	−0.1043	0.0789*	
H61	0.9693	0.0325	0.1134	0.0794*	
H222	0.6805	0.9858	0.2210	0.1264*	
H223	0.6683	0.8514	0.1473	0.1269*	
H221	0.7094	0.7849	0.2350	0.1273*	
H11	0.9322	0.1725	0.2385	0.0949*	

### Atomic displacement parameters (Å^2^)

**Table:**

	*U*^11^	*U*^22^	*U*^33^	*U*^12^	*U*^13^	*U*^23^
O1	0.0752 (11)	0.0399 (10)	0.0615 (10)	0.0001 (8)	0.0257 (9)	0.0135 (8)
O2	0.0518 (10)	0.0654 (12)	0.1058 (14)	−0.0043 (9)	0.0437 (10)	−0.0231 (10)
N1	0.0415 (10)	0.0383 (10)	0.0543 (11)	−0.0021 (8)	0.0238 (9)	0.0006 (8)
N2	0.0363 (9)	0.0547 (12)	0.0585 (12)	−0.0069 (9)	0.0171 (9)	−0.0094 (10)
C1	0.0465 (12)	0.0374 (12)	0.0636 (15)	−0.0115 (10)	0.0324 (12)	−0.0060 (11)
C2	0.0481 (13)	0.0476 (13)	0.0560 (14)	−0.0169 (11)	0.0305 (11)	−0.0092 (11)
C3	0.0607 (15)	0.0623 (16)	0.0641 (16)	−0.0240 (13)	0.0348 (13)	−0.0153 (13)
C4	0.0840 (19)	0.0658 (18)	0.0814 (19)	−0.0386 (16)	0.0579 (17)	−0.0348 (16)
C5	0.0725 (18)	0.0542 (16)	0.100 (2)	−0.0212 (14)	0.0570 (18)	−0.0257 (16)
C6	0.0564 (13)	0.0436 (13)	0.0811 (17)	−0.0112 (12)	0.0401 (13)	−0.0116 (13)
C7	0.0296 (10)	0.0354 (11)	0.0507 (12)	−0.0019 (8)	0.0187 (9)	0.0036 (9)
C8	0.0369 (10)	0.0366 (11)	0.0494 (12)	−0.0043 (9)	0.0216 (9)	0.0028 (10)
C9	0.0388 (11)	0.0449 (13)	0.0442 (12)	−0.0031 (10)	0.0171 (9)	0.0070 (10)
C10	0.0321 (10)	0.0391 (12)	0.0441 (12)	−0.0001 (9)	0.0123 (9)	0.0076 (9)
C11	0.0426 (12)	0.0419 (14)	0.0816 (17)	−0.0022 (10)	0.0307 (12)	0.0095 (12)
C12	0.0417 (12)	0.0384 (13)	0.0939 (19)	−0.0027 (10)	0.0305 (13)	−0.0044 (13)
C13	0.0370 (12)	0.0511 (14)	0.0664 (15)	0.0017 (11)	0.0211 (11)	−0.0061 (12)
C14	0.0436 (12)	0.0480 (14)	0.0668 (15)	−0.0064 (11)	0.0304 (11)	−0.0008 (12)
C15	0.0457 (12)	0.0391 (12)	0.0596 (14)	−0.0059 (10)	0.0262 (11)	0.0006 (11)
C16	0.0296 (10)	0.0379 (11)	0.0459 (12)	0.0015 (8)	0.0161 (9)	0.0061 (9)
C17	0.0392 (11)	0.0400 (13)	0.0526 (13)	0.0036 (9)	0.0206 (10)	0.0063 (10)
C18	0.0472 (13)	0.0501 (13)	0.0550 (14)	0.0061 (11)	0.0242 (11)	−0.0012 (11)
C19	0.0447 (12)	0.0661 (16)	0.0488 (13)	0.0071 (12)	0.0217 (11)	0.0046 (12)
C20	0.0476 (13)	0.0612 (15)	0.0501 (13)	0.0048 (12)	0.0221 (11)	0.0162 (12)
C21	0.0369 (11)	0.0418 (12)	0.0544 (13)	0.0019 (10)	0.0182 (10)	0.0107 (11)
C22	0.0595 (15)	0.087 (2)	0.097 (2)	−0.0058 (16)	0.0485 (16)	−0.0176 (18)

### Geometric parameters (Å, °)

**Table:**

O1—C21	1.353 (2)	C9—H91	1.002
O1—H11	0.867	C10—C11	1.392 (3)
O2—C13	1.376 (3)	C10—C15	1.378 (3)
O2—C22	1.440 (3)	C11—C12	1.374 (3)
N1—C1	1.411 (3)	C11—H111	0.975
N1—C7	1.297 (3)	C12—C13	1.389 (3)
N2—C2	1.405 (3)	C12—H121	0.964
N2—C9	1.470 (3)	C13—C14	1.371 (3)
N2—H21	0.877	C14—C15	1.397 (3)
C1—C2	1.409 (3)	C14—H141	0.972
C1—C6	1.393 (3)	C15—H151	0.986
C2—C3	1.395 (3)	C16—C17	1.405 (3)
C3—C4	1.391 (3)	C16—C21	1.409 (3)
C3—H31	0.960	C17—C18	1.375 (3)
C4—C5	1.384 (4)	C17—H171	0.976
C4—H41	0.956	C18—C19	1.381 (3)
C5—C6	1.370 (3)	C18—H181	0.968
C5—H51	0.960	C19—C20	1.371 (3)
C6—H61	0.970	C19—H191	0.993
C7—C8	1.505 (3)	C20—C21	1.396 (3)
C7—C16	1.463 (3)	C20—H201	0.953
C8—C9	1.533 (3)	C22—H222	0.997
C8—H82	0.999	C22—H223	0.985
C8—H81	0.987	C22—H221	0.995
C9—C10	1.515 (3)		
			
C21—O1—H11	108.1	C11—C10—C15	117.3 (2)
C13—O2—C22	116.84 (19)	C10—C11—C12	122.0 (2)
C1—N1—C7	120.97 (18)	C10—C11—H111	117.5
C2—N2—C9	121.12 (16)	C12—C11—H111	120.5
C2—N2—H21	117.0	C11—C12—C13	119.7 (2)
C9—N2—H21	117.0	C11—C12—H121	120.9
N1—C1—C2	122.16 (19)	C13—C12—H121	119.3
N1—C1—C6	117.7 (2)	C12—C13—O2	115.8 (2)
C2—C1—C6	119.7 (2)	C12—C13—C14	119.6 (2)
C1—C2—N2	120.6 (2)	O2—C13—C14	124.6 (2)
C1—C2—C3	118.5 (2)	C13—C14—C15	119.8 (2)
N2—C2—C3	120.6 (2)	C13—C14—H141	120.1
C2—C3—C4	120.4 (3)	C15—C14—H141	120.1
C2—C3—H31	118.6	C14—C15—C10	121.6 (2)
C4—C3—H31	120.9	C14—C15—H151	119.3
C3—C4—C5	120.6 (2)	C10—C15—H151	119.2
C3—C4—H41	119.9	C7—C16—C17	122.07 (19)
C5—C4—H41	119.5	C7—C16—C21	120.80 (19)
C4—C5—C6	119.4 (3)	C17—C16—C21	117.1 (2)
C4—C5—H51	120.6	C16—C17—C18	122.2 (2)
C6—C5—H51	120.0	C16—C17—H171	118.5
C1—C6—C5	121.3 (3)	C18—C17—H171	119.4
C1—C6—H61	118.5	C17—C18—C19	119.7 (2)
C5—C6—H61	120.2	C17—C18—H181	120.2
N1—C7—C8	119.71 (19)	C19—C18—H181	120.2
N1—C7—C16	118.03 (19)	C18—C19—C20	120.0 (2)
C8—C7—C16	122.26 (18)	C18—C19—H191	120.2
C7—C8—C9	112.03 (16)	C20—C19—H191	119.7
C7—C8—H82	108.3	C19—C20—C21	121.0 (2)
C9—C8—H82	107.3	C19—C20—H201	121.4
C7—C8—H81	110.6	C21—C20—H201	117.5
C9—C8—H81	108.3	C16—C21—C20	120.0 (2)
H82—C8—H81	110.3	C16—C21—O1	122.1 (2)
C8—C9—N2	109.30 (17)	C20—C21—O1	117.9 (2)
C8—C9—C10	111.76 (17)	O2—C22—H222	105.8
N2—C9—C10	111.18 (16)	O2—C22—H223	107.2
C8—C9—H91	107.4	H222—C22—H223	112.5
N2—C9—H91	109.2	O2—C22—H221	109.1
C10—C9—H91	107.8	H222—C22—H221	111.4
C9—C10—C11	118.86 (19)	H223—C22—H221	110.6
C9—C10—C15	123.85 (19)		

### Hydrogen-bond geometry (Å, °)

**Table:**

*D*—H···*A*	*D*—H	H···*A*	*D*···*A*	*D*—H···*A*
O1—H11···N1	0.87	1.74	2.523 (3)	148
